# A comparison of demographic, epidemiological and clinical characteristics of hospital influenza-related viral pneumonia patients

**DOI:** 10.1186/s12879-021-06485-x

**Published:** 2021-09-25

**Authors:** Bin Fu, Zhengjie Wu, Lingtong Huang, Zhaohui Chai, Peidong Zheng, Qinmiao Sun, Silan Gu, Qiaomai Xu, Haiting Feng, Lingling Tang

**Affiliations:** 1 Department of Infectious Diseases, Hangzhou TCM Hospital Affiliated to Zhejiang Chinese Medical University, Hangzhou, 310007 People’s Republic of China; 2 State Key Laboratory for Diagnosis and Treatment of Infectious Disease, National Clinical Research Center for Infectious Diseases, Collaborative Innovation Centre for Diagnosis and Treatment of Infectious Diseases, The First Affiliated Hospital, College of Medicine, Zhejiang University, No.79 Qingchun Road, Hangzhou, 310003 Zhejiang People’s Republic of China; 3 Department of Critical Care Medicine, The First Affiliated Hospital, College of Medicine, Zhejiang University, Hangzhou, 310003 People’s Republic of China; 4 Department of Neurosurgery, The First Affiliated Hospital, College of Medicine, Zhejiang University, Hangzhou, 310003 People’s Republic of China; 5 Department of Dermatology, The First Affiliated Hospital, College of Medicine, Zhejiang University, Hangzhou, 310003 People’s Republic of China; 6 Department of Nosocomial Infection, The First Affiliated Hospital, College of Medicine, Zhejiang University, Hangzhou, 310003 People’s Republic of China; 7 Shulan(Hangzhou) Hospital Affiliated to Zhejiang Shuren University Shulan International Medical College, Hangzhou, 310006 People’s Republic of China

**Keywords:** Human influenza A, Influenza B, Avian-origin influenza H7N9, Viral pneumonia, Severe cases

## Abstract

**Background:**

Through the comparison of the demographic, epidemiological, and clinical characteristics of hospital human influenza (influenza A (H1N1) pdm09, H3N2, and B)-related and hospitalized avian-origin influenza A (H7N9)-related viral pneumonia patients, find the different between them.

**Methods:**

A retrospective study was conducted in hospitalized influenza-related viral pneumonia patients.

**Results:**

Human influenza A-related patients in the 35–49-year-old group were more than those with B pneumonia patients (p = 0.027), and relatively less in the ≥ 65-year-old group than B pneumonia patients (p = 0.079). The proportion of comorbid condition to human influenza A pneumonia was 58%, lower than B pneumonia and H7N9 pneumonia patients (78% vs. 77.8%; p = 0.013). The proportion of invasive mechanical ventilation (IMV), lymphocytopenia, elevated lactate dehydrogenase to hospitalized human influenza A-related viral pneumonia patients was higher than B pneumonia patients (p < 0.05), but lower than H7N9 pneumonia patients (p < 0.05). In the multivariate analysis, pulmonary consolidation (odds ratio (OR): 13.67; 95% confidence interval (CI) 1.54–121.12; p = 0.019) and positive bacterial culture (sputum) (OR: 7.71; 95% CI 2.48–24.03; p < 0.001) were independently associated with IMV, while shock (OR: 13.16; 95% CI 2.06–84.07; p = 0.006), white blood cell count > 10,000/mm^3^ (OR: 7.22; 95% CI 1.47–35.58; p = 0.015) and positive bacterial culture(blood or sputum) (OR: 6.27; 95% CI 1.36–28.85; p = 0.018) were independently associated with death in the three types hospitalized influenza-related viral pneumonia patients.

**Conclusions:**

Hospital influenza B-related viral pneumonia mainly affects the elderly and people with underlying diseases, while human influenza A pneumonia mainly affects the young adults; however, the mortality was similar. The hospitalized human influenza A-related viral pneumonia patients was severer than B pneumonia patients, but milder than H7N9 pneumonia patients. Pulmonary consolidation and positive bacterial culture (sputum) were independently associated with IMV, while shock, white blood cell count > 10,000/mm^3^, and positive bacterial culture (blood or sputum) were independently associated with death to three types hospitalized influenza-related viral pneumonia patients.

**Supplementary Information:**

The online version contains supplementary material available at 10.1186/s12879-021-06485-x.

## Background

Influenza causes considerable morbidity during each annual influenza season. In April 2009, the influenza A (H1N1) pdm09 (pH1N1) virus emerged in Mexico and the USA and spread globally [1]. Since the first report of pneumonia that was caused by the pH1N1 virus in Mexico [2], severe cases have been documented worldwide. Influenza B virus has been classically considered less pathogenic than influenza A virus in adults and mostly responsible for mild respiratory infections, while severe illness and poor prognosis have been associated with bacterial coinfection [3, 4]. On March 30, 2013, three individuals with severe pneumonia were found to be infected with a novel avian-origin influenza A (H7N9) virus that had not been detected in humans and animals previously. As of May 9, 2013, 131 laboratory-confirmed cases were reported, including 32 deaths [5], and the majority of these patients were reported in China.

The comparison of the demographic, epidemiological, and clinical characteristics of hospital human influenza (influenza A (H1N1) pdm09, H3N2, and B)-related and hospitalized avian-origin influenza A (H7N9)-related viral pneumonia patients has not been reported. 2017–2018 influenza season was dominated by pH1N1 and B viruses along with co-circulation of H3N2 virus, this season provided a unique opportunity to directly compare the demographic, epidemiological, and clinical characteristics of hospitalized human influenza A (pH1N1 and H3N2)-related viral pneumonia patients to the hospital influenza B-related viral pneumonia patients. Also, we compared the demographic, epidemiological, and clinical characteristics of these hospitalized human influenza (pH1N1, H3N2, and B)-related viral pneumonia patients to the hospital H7N9-related viral pneumonia patients in 2017, and aimed to discover the factors independently associated with invasive mechanical ventilation (IMV) or death among these three types hospitalized influenza-related viral pneumonia patients.

## Methods

### Study design and study population

A retrospective study was conducted from November 1, 2017 to March 31, 2018 at the First Affiliated Hospital, College of Medicine, Zhejiang University, China. All patients, > 14 years of age and with hospital stay > 24 h with confirmed human influenza A or B-related viral pneumonia were included in this study. In addition to above factors, those with confirmed H7N9-related viral pneumonia from January 1, 2017 to May 3, 2017 were also included. This study was approved by the Ethical Board of the hospital.

This retrospective study evaluated the reverse transcriptase-polymerase chain reaction (RT-PCR) tests and computed tomography results daily in hospitalized patients with confirmed human influenza A- or B-related viral pneumonia or H7N9-related viral pneumonia. These cases were assessed by an Infectious Disease doctor and nasopharyngeal swabs (NPS) or sputum specimens were collected within the first 24 h of hospitalization and processed immediately. Influenza-related viral pneumonia was defined as confirmed human influenza A or B or H7N9 virus infection and the presence of a new infiltrate on computed tomography plus fever or respiratory symptoms. All radiological evaluations were performed by two radiologists who were blinded to the clinical information.

### Data collection and definitions

Clinical data were acquired from electronic medical records. The following variables were recorded: demographics, comorbid conditions, current smoking status, alcohol abuse, pregnancy, body mass index (BMI), clinical feature, laboratory data, radiographic finding, complication, treatment, and clinical outcome. The definitions of obesity, current smoking status, alcohol abuse, confirmed human influenza A or B, confirmed H7N9, lymphocytopenia, thrombocytopenia, rhabdomyolysis, acute kidney injury, immunosuppression, early antiviral therapy, exposure to live poultry and severe cases were provided in Additional file 1.

### Statistical analysis

Categorical variables were compared by the Chi-square or Fisher’s exact test. Continuous variables were compared by the t-test or the Mann–Whitney test. The multivariate logistic regression analysis of factors potentially associated with IMV or in-hospital mortality included the variables that were significant in the univariate analysis and clinically important variables. Statistical significance was established at p-value < 0.05. All statistical analyses were performed using SPSS v.18. (SPSS Inc., Chicago, IL, USA).

## Results

### Demographic and epidemiological characteristics

From November 1, 2017 to March 31, 2018, a total of 4297 cases of laboratory-confirmed human influenza A and B viral infection were reported in our hospital. Of these, 2335 cases were human influenza A (about 85% were pH1N1 and 15% were H3N2), 1880 cases were influenza B, and 82 cases were infected with both viruses. Finally, 138 cases of hospitalized human influenza A-related viral pneumonia and 59 cases of hospitalized influenza B-related viral pneumonia were included in this study. From January 1, 2017 to May 3, 2017, a total of 18 cases of hospitalized H7N9-related viral pneumonia were also included in the current study. The median age of hospitalized human influenza A-related viral pneumonia patients was lower than those with B pneumonia (57 years (interquartile range (IQR), 45.8–66.3) vs. 62 years (IQR, 53–74); p = 0.034). In the 35–49-year-old age group, the proportion of hospitalized human influenza A-related viral pneumonia patients was 23.9%, which was higher than B pneumonia patients (10.2%; p = 0.027). In the ≥ 65-year-old group, the proportion of hospitalized human influenza A-related viral pneumonia patients was 32.6%, which was relatively lower than B pneumonia patients (45.8%; p = 0.079; Table 1). The proportion of comorbid conditions in hospitalized human influenza A-related viral pneumonia patients was 58%, which was lower than the B pneumonia patients and hospitalized H7N9 pneumonia patients (78% vs. 77.8%; p = 0.013). Interestingly, 66.7% of hospitalized H7N9-related viral pneumonia patients were exposed to live poultry.

**Table 1:** Comparison of demographic and epidemiological characteristics of three types hospitalized influenza-related viral pneumonia patients

Characteristics	B (n = 59)	A (n = 138)	H7N9 (n = 18)	p-value among	
	(a)	(b)	(c)	(a) and (b)	(a), (b), and (c)
Demographic data					
Age (y)					
Median (IQR)	62 (53–74)	57 (45.8–66.3)	60 (43.3–68)	0.034	0.095
Age subgroup (y), no. (%)					
14–34	7 (11.9)	12 (8.7)	1 (5.6)	0.49	0.752
35–49	6 (10.2)	33 (23.9)	4 (22.2)	0.027	0.08
50–64	19 (32.2)	48 (34.8)	7 (38.9)	0.726	0.863
≥ 65	27 (45.8)	45 (32.6)	6 (33.3)	0.079	0.205
Female, no. (%)	22 (37.3)	49 (35.5)	4 (22.2)	0.812	0.486
Comorbid conditions, no. (%)					
Any one condition	46 (78.0)	80 (58.0)	14 (77.8)	0.007	0.013
Chronic pulmonary disease	7 (11.9)	20 (14.5)	2 (11.1)	0.623	0.949
Diabetes	5 (8.5)	24 (17.4)	2 (11.1)	0.106	0.289
Hypertension	21 (35.6)	54 (39.1)	8 (44.4)	0.64	0.778
Chronic renal diseases	5(8.5)	7 (5.1)	2 (11.1)	0.556	0.327
Chronic liver diseases	7 (11.9)	15 (10.9)	5 (27.8)	0.839	0.132
Chronic cardiovascular disease	5 (8.5)	25 (18.1)	2 (11.1)	0.085	0.224
Chronic neurological disease	4 (6.8)	5 (3.6)	1 (5.6)	0.549	0.495
Hematological diseases	10 (16.9)	12 (8.7)	0 (0)	0.092	0.085
Immunosuppression	7 (11.9)	11 (8)	0 (0)	0.385	0.305
Pregnancy	0 (0)	1 (0.7)	0 (0)	1	1
Current smoker	9 (15.3)	36 (26.1)	7 (38.9)	0.097	0.076
Alcohol abuse	8 (13.6)	27 (19.6)	3 (16.7)	0.312	0.633
BMI, no. (%)					
Obesity + morbid obesity	0 (0)	7 (5.1)	0 (0)	0.105	0.229
Exposure to live poultry in previous 14 days, no. (%)	NA	NA	12 (66.7)		

Body mass index: For human influenza A, there were 40 miss dates; for B, there were 18 miss dates; for H7N9, there were 3 miss dates

*IQR* interquartile range

### Clinical characteristics and diagnostic findings

The most common symptoms of the three types of hospital influenza-related viral pneumonia patients were fever, cough, sputum production, shortness of breath, and fatigue (Table 2).

**Table 2:** Comparison of clinical characteristics of three types hospitalized influenza-related viral pneumonia patients

Characteristics	B (n = 59)	A (n = 138	H7N9 (n = 18)	p-value among	
	(a)	(b)	(c)	(a) and (b)	(a), (b), and (c)
Clinical features					
Fever					
Any no. (%)	54 (91.5	23 (89.1)	17 (94.4)	0.62	0.819
Maximal temperature subgroup, no (%) (°C)					
37.3–38.0	11 (20.4)	12 (10.6)	3 (18.8)	0.046	0.173
38.1–39.0	25 (46.3)	46 (40.7)	6 (37.5)	0.226	0.734
> 39.0	18 (33.3)	55 (48.7)	7 (43.8)	0.213	0.174
Cough	52 (88.1)	120 (87)	17 (94.4)	0.82	0.845
Sputum production	50 (84.7)	106 (76.8)	16 (88.9)	0.209	0.348
Shortness of breath	29 (49.2)	83 (60.1)	12 (66.7)	0.154	0.26
Fatigue/weakness	34 (57.6)	67 (48.6)	6 (33.3)	0.243	0.175
Myalgia	4 (6.8)	16 (11.6)	1 (5.6)	0.305	0.617
Laboratory findings					
White blood cells count/mm^3^, median (IQR)	6900 (4300–12,600)	7000 (4300–9550)	4400 (3475–5625)	0.407	0.008
Subgroup, no. (%)					
> 10,000/mm^3^	17 (28.8)	32 (23.2)	1 (5.6)	0.403	0.12
< 4000/mm^3^	13 (22)	28 (20.3)	7 (38.9)	0.782	0.224
Lymphocytes count/mm^3^, median (IQR)	1020 (550–1690)	745 (448–1208)	425 (375–625)	0.34	0.001
Lymphocytopenia, no. (%)	41 (69.5)	115 (83.3)	18 (100)	0.028	0.006
Hemoglobulin—g/dL, median (IQR)	117 (95–130)	124 (104–139)	117 (109.3–129)	0.018	0.058
Platelets count/mm^3^, median (IQR)	159,000 (107,000–251,000)	164,500 (103,300–250,000)	139,000 (93,800–173,500)	0.842	0.335
Thrombocytopenia, no. (%)	26 (44.1)	60 (43.5)	11 (61.1)	0.939	0.361
C-reactive protein > 10 mg/L, no. (%)	46 (82.1)	115 (83.9)	16 (88.9)	0.76	0.836
Procalcitonin > 0.5 ng/mL, no. (%)	8 (25.8)	34 (31.8)	8 (44.4)	0.525	0.401
Alanine aminotransferase > 40 U/L, no. (%)	15 (25.9)	50 (36.5)	8 (44.4)	0.15	0.229
Aspartate aminotransferase > 40 U/L, no. (%)	15 (25.9)	64 (46.7)	14 (77.8)	0.007	< 0.001
Total bilirubin > 21 μmol/L, no. (%)	5 (8.6)	10 (7.3)	0 (0)	0.982	0.585
Creatinine > 104 μmol/L (1.5 mg/dL), no. (%)	12 (20.3)	19 (13.9)	2 (18)	0.255	0.479
Lactate dehydrogenase > 250 U/L, no. (%)	26 (51)	91 (67.9)	17 (100)	0.033	0.001
Creatine kinase > 200 U/L, no. (%)	5 (9.8)	24 (17.9)	13 (76.5)	0.175	< 0.001
PaO_2_:FiO_2_ (mmHg), median (IQR)	227.5 (173.5–302.8)	214.1 (136.6–293.2)	171.6 (137.5–233.3)	0.214	0.118
PaO_2_:FiO_2_ ≤ 300 mmHg, no. (%)	23 (71.9)	75 (78.1)	16 (88.9)	0.47	0.41
D-dimer > 700 μg/L, no. (%)	35 (62.5)	104 (76.5)	17 (94.4)	0.049	0.016
Positive culture (blood or sputum) on presentation or during hospitalization, no. (%)					
Bacterial	10 (21.3)	24 (21.1)	11 (61.1)	0.975	0.002
Fungal	3 (6.4)	18 (15.8)	6 (33.3)	0.107	0.028
Radiographic findings					
Computed tomography consistent with pneumonia at admission, no. (%)					
Involvement of both lungs	52 (88.1)	121 (87.7)	16 (88.9)	0.929	1
Ground-glass opacity	12 (20.3)	49 (35.5)	10 (55.6)	0.035	0.012
Consolidation	29 (49.2)	88 (63.8)	18 (100)	0.056	< 0.001
CURB-65 score ≥ 2	18 (30.5)	38 (27.5)	6 (33.3)	0.672	0.798

Maximal temperature: For human influenza A, there were 25 miss dates; for B, there were 5 miss dates; for H7N9, there were 2 miss dates

C-reactive protein: For human influenza A, there were 2 miss dates; for B, there were 2 miss dates

Procalcitonin: For human influenza A, there were 32 miss dates; for B, there were 28 miss dates

Alanine aminotransferase: For human influenza A, there were 2 miss dates; for B, there was 1 miss date

Aspartate aminotransferase: For human influenza A, there were 2 miss dates; for B, there was 1 miss date

Total bilirubin: For human influenza A, there were 2 miss dates; for B, there was 1 miss date

Creatinine: For human influenza A, there were 2 miss dates

Lactate dehydrogenase: For human influenza A, there were 5 miss dates; for B, there were 8 miss dates; for H7N9, there were 2 miss dates

Creatine kinase: For human influenza A, there were 5 miss dates; for B, there were 8 miss dates; for H7N9, there were 2 miss dates

Partial pressure arterial oxygen/fraction of inspired oxygen: For human influenza A, there were 43 miss dates; for B, there were 27 miss dates

D-Dimer: For human influenza A, there were 3 miss dates; for B, there were 3 miss dates

Positive culture (blood or sputum) on presentation or during hospitalization (bacterial): For human influenza A, there were 25 miss dates; for B, there were 12 miss dates

Positive culture (blood or sputum) on presentation or during hospitalization (fungal): For human influenza A, there were 25 miss dates; for B, there were 12 miss dates

*PaO*_*2*_*:FiO*_*2*_ partial pressure arterial oxygen/fraction of inspired oxygen, *IQR* interquartile range

At the time of admission, the proportion of lymphocytopenia in hospitalized human influenza A-related viral pneumonia patients was 83.3%, higher than B pneumonia patients (69.5%; p = 0.028). The proportion of elevated aspartate aminotransferase (AST), lactate dehydrogenase (LDH) in hospitalized human influenza A-related viral pneumonia patients was higher than that of B pneumonia patients (46.7% vs. 25.9%, p = 0.007; 67.9% vs. 51%, p = 0.033). The median value of lymphocyte count in hospitalized H7N9-related viral pneumonia patients was 425/mm^3^ (IQR: 375–625), lower than human influenza A and B pneumonia patients (745/mm^3^ (IQR: 448–1208) vs. 1020/mm^3^ (IQR: 550–1690); p = 0.001). The proportion of lymphocytopenia in hospitalized H7N9-related viral pneumonia patients was 100.0%, higher than human influenza A and B pneumonia patients (83.3% vs. 69.5%; p = 0.006). The proportion of elevated AST, creatine kinase (CK), DD, and LDH in hospitalized H7N9-related viral pneumonia patients was higher than human influenza A and B pneumonia patients (77.8% vs. 46.7% vs. 25.9%, p < 0.001; 76.5% vs. 17.9% vs. 9.8%, p < 0.001; 94.4% vs. 76.5% vs. 62.5%, p = 0.016; 100% vs. 67.9% vs. 51%, p = 0.001). The proportion of positive culture (blood or sputum) on presentation or during hospitalization in the hospitalized H7N9-related viral pneumonia patients (bacterial) was 61.1%, higher than the human influenza A and B pneumonia patients (21.1% vs. 21.3%; p = 0.002).

### Complications, treatment, and clinical outcomes

In the current study, the proportion of intensive care unit (ICU) admission in hospitalized human influenza A-related viral pneumonia patients was 19.6%, which higher than 6.8% of B pneumonia patients (p = 0.024). The proportion of IMV in hospitalized human influenza A-related viral pneumonia patients was 16.7%, which also higher than that in B pneumonia patients 3.4% (p = 0.01) (Table 3). The proportion of IMV in hospitalized H7N9-related viral pneumonia patients was 38.9%, which was higher than that in human influenza A and B pneumonia patients (16.7% vs. 3.4%; p = 0.001). The median value of hospital expense of hospitalized H7N9-related viral pneumonia patients was 66,095.6 Yuan (IQR, 42,450.6–129,574.2), which was higher than that of human influenza A and B pneumonia patients (IQR, 25,642.3 Yuan (13,184.5–53,805.7) vs. 18,316.8 Yuan (IQR, 11,111.1–35,750.5); p < 0.001). There was no difference in death to three types of hospitalized influenza-related viral pneumonia patients.

**Table 3:** Comparison of complications, treatment, and clinical outcomes of three types hospitalized influenza-related viral pneumonia patients

Characteristics	B (n = 59)	A (n = 138)	H7N9 (n = 18)	p-value among	
	(a)	(b)	(c)	(a) and (b)	(a), (b), and (c)
Complications	7 (12)	16 (12)	4 (22.2)	0.957	0.411
Shock	1 (1.7)	9 (6.5)	1 (5.6)	0.289	0.315
Acute kidney injury	5 (8.5)	11 (8)	2 (11.1)	1	0.793
Rhabdomyolysis	1 (1.7)	1 (0.7)	1 (5.6)	0.51	0.147
Treatment					
Oxygen therapy, no. (%)	44 (74.6)	101 (73.2)	18 (100)	0.84	0.022
Antiviral treatment, no. (%)	52 (88)	152 (93)	18 (100)	0.72	0.388
Oseltamivir	36 (69.2)	70 (56.5)	0 (0)	0.114	< 0.001
Peramivir	9 (17.3)	32 (25.8)	0 (0)	0.224	0.02
Both	7 (13.5)	22 (17.7)	18 (100)	0.485	< 0.001
Timing from onset of illness to administration of antiviral therapy, median (IQR) (days)	6 (3–14)	7 (5–10.8)	4 (3–8.3)	0.797	0.089
0–2	9 (17.6)	14 (11.3)	1 (18)	0.258	0.384
3–5	12 (23.5)	31 (25)	57 (34.8)	0.837	0.091
≥ 6	30 (58.8)	79 (63.7)	8 (44.4)	0.544	0.281
Timing from administration of antiviral therapy to virus negative, median (IQR)	11 (6–17.75)	10 (6–14.5)	5 (2.8–10)	0.26	0.007
Admission to ICU, no. (%)	4 (6.8)	27 (19.6)	15 (83.3)	0.024	0.054
Mechanical ventilation					
Noninvasive	6 (10.2)	14 (10.1)	0 (0)	0.996	0.528
Invasive	2 (3.4)	23 (16.7)	7 (38.9)	0.001	0.001
Extracorporeal membrane oxygenation	0 (0)	2 (1.4)	2 (11.1)	1	0.048
Blood purification	4 (6.8)	3 (2.2)	2 (11.1)	0.2	0.072
Antibiotics	56 (94.9)	134 (97.1)	17 (94.4)	0.430	0.416
Antifungal	22 (37.3)	42 (30.4)	13 (72.2)	0.347	0.002
Glucocorticoids	24 (41)	77 (56)	18 (100)	0.052	< 0.001
Clinical outcome					
Death	5 (8.5)	13 (9.4)	0 (0)	0.833	0.542
Total hospitalization expenses (Yuan), median (IQR)	18,316.8 (11,111.1–35,750.5)	25,642.3 (13,184.5–53,805.7)	66,095.6 (42,450.6–129,574.2)	0.094	< 0.001

Timing from onset of illness to administration of antiviral therapy: For human influenza A, there were 14 miss dates; for B, there were 8 miss dates

Timing from administration of antiviral therapy to virus negative: For human influenza A, there were 73 miss dates; for B, there were 39 miss dates

Total hospitalization expenses: For human influenza A, there were 14 miss dates; for B, there were 6 miss dates

*ICU* intensive care unit, *IQR* interquartile range

In the treatment, the rate of glucocorticoid use in hospitalized H7N9-related viral pneumonia patients was 100%, which was higher than human influenza A and B pneumonia patients (55.8% vs. 40.7%; p < 0.001). All the hospitalized human influenza A-related viral pneumonia patients and hospitalized influenza B-related viral pneumonia patients were treated with oseltamivir or peramivir or both, and all the hospitalized H7N9-related viral pneumonia patients were treated with a combination of oseltamivir with peramivir. However, the time from onset of illness to administration of antiviral therapy in hospitalized H7N9-related viral pneumonia patients was 4 days (IQR, 3–8.3), which was relatively shorter than that of human influenza A and B pneumonia patients (7 days (IQR, 5–10.8) vs. 6 days (range, 3–14); p = 0.089). The time of administration of antiviral therapy to virus-negative time of hospitalized H7N9-related viral pneumonia patients was 5.5 days (IQR, 2.8–10), shorter than human influenza A and B pneumonia patients (10 days (IQR, 6–14.5) *vs*. 11 days (IQR, 6–17.75); p = 0.007).

### Factors associated with IMV due to three types hospitalized influenza-related viral pneumonia patients

In the univariate analysis, the three types hospitalized influenza-related viral pneumonia patients with IMV had significantly higher neutrophil percentage, C-reactive protein, AST, DD, blood urea nitrogen, pro-B-type natriuretic peptides, and partial pressure arterial oxygen/fraction of inspired oxygen (PaO_2_:FiO_2_) levels than the three types hospitalized influenza-related viral pneumonia patients without IMV. Furthermore, the proportion of patients with shortness of breaths, lymphocytopenia, elevated procalcitonin, AST, CK, DD, and LDH, positive bacterial culture (sputum) on presentation or during hospitalization, positive bacterial culture (blood or sputum) on presentation or during hospitalization, CURB-65 score ≥ 2, bilateral pneumonia, and pulmonary consolidation in the three types of hospitalized influenza-related viral pneumonia patients with IMV was significantly higher than those without IMV, while the lymphocyte count in the three types of pneumonia patients with IMV was lower than those without IMV (Additional file 2: Table S1). In the multivariate analysis, pulmonary consolidation (odds ratio (OR): 13.67; 95% confidence interval (CI) 1.54–121.12; p = 0.019) and positive bacterial culture (sputum) at the time of presentation or during hospitalization (OR: 7.71; 95% CI 2.48–24.03; p < 0.001) were independently associated with IMV in the three types hospitalized influenza-related viral pneumonia patients (Additional file 4: Table S3).

### Factors associated with death due to the three types hospitalized influenza-related viral pneumonia patients

In the univariate analysis, the three types of hospitalized influenza-related viral pneumonia patients who die in the hospital had significantly higher neutrophil percentage, hemoglobin, blood platelet counts, C-reactive protein, DD, blood urea nitrogen, pro-B-type natriuretic peptides, and PaO_2_:FiO_2_ levels than the survivors. Also, the proportion of elevated white blood cell count and procalcitonin, positive bacterial culture (sputum) on presentation or during hospitalization, CURB-65 score ≥ 2, IMV, shock to the three types hospitalized influenza-related viral pneumonia patients who die in the hospital was significantly higher than that of the survivors (Additional file 3: Table S2). In the multivariate analysis, shock (OR: 13.16; 95% CI 2.06–84.07; p = 0.006), white blood cell count > 10,000/mm^3^ (OR: 7.22; 95% CI 1.47–35.58; p = 0.015) and positive bacterial culture (blood or sputum) on presentation or during hospitalization (OR: 6.27; 95% CI 1.36–28.85; p = 0.018) were independently associated with death in the three types hospitalized influenza-related viral pneumonia patients (Table 4).

**Table 4:** Multivariate analysis of factors associated with death due to three types hospitalized influenza-related viral pneumonia patients

Variables	OR (95% CI)	p-value
White blood cell count > 10,000/mm^3^	7.22 (1.47–35.58)	0.015
Hemoglobulin (g/dL)		0.1
Platelets count/mm^3^		0.851
C-reactive protein (mg/L)		0.772
Procalcitonin > 0.5 ng/mL		0.621
D-dimer > 2100 mg/L		0.387
Pro-B-type natriuretic peptides (pg/mL)		0.231
Positive bacterial culture (blood or sputum) on presentation or during hospitalization	6.27 (1.36–28.85)	0.018
Computed tomography consistent with pulmonary consolidation at admission		0.427
Shock^a^	13.16 (2.06–84.07)	0.006

*OR* odds ratio, *CI* confidence interval

Pro-B-type natriuretic peptides: For human influenza A, there were 62 miss dates; for B, there were 42 miss dates; for H7N9, there were 11 miss dates

^a^Shock at any point during the illness

## Discussion

This was the first study that compared the demographic, epidemiological, and clinical characteristics of hospitalized human influenza-related viral pneumonia patients with H7N9 pneumonia patients. The study revealed that the hospitalized human influenza A-related viral pneumonia patients were younger than those with B pneumonia, and the infection was primarily detected in young adults. On the other hand, the influenza B-related viral pneumonia was common in the elderly, usually with comorbid conditions. Hospitalized human influenza A-related viral pneumonia patients was severer than B pneumonia patients, but milder than H7N9 pneumonia patients.

The proportion of patients who have at least one comorbid condition in those with human influenza A-related viral pneumonia was lower than those with B pneumonia and H7N9 pneumonia patients. Moreover, the average age of hospitalized human influenza A-related viral pneumonia patients was lower than that of B pneumonia patients, and the proportion of the hospitalized human influenza A-related viral pneumonia patients in the 35–49 age group was more than that of the B pneumonia patients, and the proportion in the ≥ 65 years age group was relatively less than that of the B pneumonia patients. Some studies have reported that influenza B mainly affects the elderly and people with underlying diseases [6, 7]; however, pH1N1 mainly affects young adults, similar to our research. Early-life exposure to an antigenically related virus, i.e., the A (H1N1) strain that circulated after the 1918 pandemic and prior to the 1957 A (H2N2) pandemic might mitigate the severity for the pH1N1 strain in older individuals; this phenomenon explained the survival of elderly in the pandemic. In China, the old people retire at 60 years of age and have more opportunities to shop in live-animal markets, therefore more likely to be exposed to the live-poultry, it can partially explain why the H7N9 patients have more comorbid conditions.

In the aspect of clinical characteristics and diagnostic findings, CK was designated as a biomarker of severity in pH1N1 infection; the elevation of CK was associated with complications, increased length of ICU stay, and healthcare resources [8]. Another study included 155 hospitalized adult patients with pH1N1; LDH was an independent risk factor of hospital death as assessed by multivariate logistic regression analysis [9]. The elevation of DD may be caused by the embolism of the small pulmonary vessels, then the pulmonary ventilation and blood flow ratio became abnormal, and the blood oxygen saturation declined. Finally, the rate of mortality was elevated; however, this hypothesis needs further substantiation. A lymphocyte count of < 300 lymphocytes/μL was observed in a subgroup of patients with poor outcome in a study encompassing 239 inpatients with confirmed influenza virus infection [10]. Thus, we can conclude that the proportion of elevated CK, LDH, DD, and lymphocytopenia was positively relative to the severity of influenza. In this study, the elevated LDH and lymphocytopenia in hospitalized human influenza A-related viral pneumonia patients was higher than that in B pneumonia patients, and the elevated CK, LDH, DD, and lymphocytopenia in hospitalized H7N9-related viral pneumonia patients was higher than that in human influenza A and B pneumonia patients. The proportion of ICU admission and invasive mechanical ventilation in hospitalized human influenza A-related viral pneumonia patients was higher than that in B pneumonia patients. The proportion of IMV and the median value of hospitalization expense in hospitalized H7N9-related viral pneumonia patients were higher than that in human influenza A and B pneumonia patients. Taken together, it can be concluded that the hospitalized human influenza A-related viral pneumonia patients was severer than B pneumonia patients, but milder than H7N9 pneumonia patients. A study consisting of 57 cases of H7N9 patients and 14 cases of pH1N1 patients demonstrated that the proportion of acute respiratory distress syndrome to H7N9 was much higher than that of pH1N1 [11], which was also observed in the current study. Furthermore, the proportion of ICU admission and IMV to pH1N1 was twofold higher than that of B, and no difference was detected in the mortality as reported by a study including 2791 cases of adult patients [12]. Another study reported that the mortality of adults influenza B patients was similar to that of pH1N1 patients [13]. Although all the influenza viruses infect the respiratory epithelium from the nasal passages to bronchioles, pH1N1 tends to infect pneumocytes and interalveolar macrophages, causing extensive areas of inflammation in the alveoli, which could partially explain the increased severity of pH1N1 [14–17]. Hospitalized influenza B-related viral pneumonia mainly affects the elderly and those with underlying diseases, rendering them as severe cases, such that the mortality of B pneumonia patients was elevated and similar to human influenza A pneumonia patients. Hypercytokinemia is characterized by the over-production of various proinflammatory cytokines and plays an significant role in disease severity and mortality in H5N1 patients [18]. Compared with pH1N1 infection, H7N9 virus infection tends to induce higher cytokine expression, resembling the cytokine storm observed in H5N1 infection [19], which could partially explain why the H7N9 infection was severer than pH1N1.

The World Health Organization Guidelines recommended that prompt empirical antiviral treatment should be initiated when influenza is suspected, even before laboratory results are known [20]. Improved clinical outcomes have been described among adults treated with antiviral drugs after hospitalization or up to 5 days from illness onset [21–23]. Early antiviral therapy within 2 days of illness accelerated viral shedding and reduced the mortality in patients with H7N9 viral infection [24]. Moreover, compared to early antiviral therapy, H7N9 patients with delayed antiviral therapy became severe cases [25]. Interestingly, in a study comprising of 82 cases of H7N9 virus infection, the time of onset of illness to administration of antiviral therapy was 6 days (IQR: 4–8), longer than that in the current study 4 days (IQR:3–8.3), and the administration of antiviral therapy to virus-negative time of the study was 7 days (IQR: 5–9), also longer than 5.5 days (IQR: 2.8–10) in the current study; the mortality was 34.1% [26]. Gao et al. [27] reported that the rate of glucocorticoid use of the H7N9 cases was 62.2%, lower than our study 100% and the mortality was 27%. The mortality of hospitalized H7N9-related viral pneumonia patients was much higher than that of human influenza A and B pneumonia. However, in the current study, the mortality of hospitalized H7N9-related viral pneumonia patients was lower than before and similar to human influenza A and B pneumonia. This phenomenon might be explicated by the initial antiviral time relatively early than before and the rate of glucocorticoid use for H7N9 was high. All our patients received oseltamivir with peramivir that also may reduce the mortality, although these findings need to be substantiated further. In the current study, the proportion of early antiviral therapy in the three types of hospitalized influenza-related viral pneumonia patients was similar.

Nevertheless, the present study has several limitations. First, influenza testing was ordered at the physicians’ discretion, and therefore, not all individuals with influenza were tested, or testing could be biased towards severe cases. However, this bias would not affect our main findings because influenza testing was ordered independently of the knowledge of the virus type or subtype. Second, we only included the hospitalized influenza-related viral pneumonia patients and excluded the outpatient cases and hospitalized patients without influenza-related viral pneumonia, such that it cannot reflect the whole disease spectrum. Finally, because the RT-PCR was not accurate absolutely, the real influenza-positive patients may be diagnosed as negative.

## Conclusion

In summary, hospitalized influenza B-related viral pneumonia mainly affects the elderly and individuals with underlying diseases, while human influenza A pneumonia mainly affects the young adults; however, the mortality was comparative. The hospitalized human influenza A-related viral pneumonia patients was severer than B pneumonia patients, but milder than H7N9 pneumonia patients. Pulmonary consolidation and positive bacterial culture (sputum) at the time of presentation or during hospitalization were independently associated with IMV due to three types of hospital influenza-related viral pneumonia patients. On the other hand, shock, white blood cell count > 10,000/mm^3^, and positive bacterial culture (blood or sputum) at the time of presentation or during hospitalization were associated with death in such patients.

## Supplementary Information

**Additional file 1.** Definitions of Obesity, The current smoking status, Alcohol abuse, Confirmed human influenza A or B, Comfirmed avian-origin A (H7N9), lymphocytopenia, Rhabdomyolysis, Acute kidney injury, Immunosuppression, Early antiviral therapy, The exposure to live poultry.

**Additional file 2. Table S1:** Univariate Analysis of Factors Associated with Invasive Mechanical Ventilation due to Three Types Hospitalized Influenza-related Viral Pneumonia Patients.

**Additional file 3. Table S2:** Univariate Analysis of Factors Associated with Death due to Three Types Hospitalized Influenza-related Viral Pneumonia Patients.

**Additional file 4. Table S3:** Multivariate Analysis of Factors Associated with Invasive Mechanical Ventilation due to Three Types of Hospital Influenza-related Viral Pneumonia Patients.

## Data Availability

All data generated or analysed during this study are either included in this published article and its additional information files or are available from the corresponding author on reasonable request.

## Abbreviations

IMV: Invasive mechanical ventilation
pH1N1: Influenza A (H1N1) pdm09
H7N9: Avian-origin influenza A (H7N9)
RT-PCR: Reverse transcriptase-polymerase chain reaction
NPS: Nasopharyngeal swabs
BMI: Body mass index
IQR: Interquartile range
AST: Aspartate aminotransferase
LDH: Lactate dehydrogenase
DD: D-dimer
CK: Creatine kinase
ICU: Intensive care unit
ECMO: Extracorporeal membrane oxygenation
PaO_2_:FiO_2_: Partial pressure arterial oxygen/fraction of inspired oxygen
OR: Odds ratio
CI: Confidence interval
SCr: Serum creatinine
