# Supplementary material for: A comparison of demographic, epidemiological and clinical characteristics of hospital influenza-related viral pneumonia patients
Source: BMC Infect Dis. 2021 Sep 25;21:1002. doi: 10.1186/s12879-021-06485-x (PMC8466655; doi:10.1186/s12879-021-06485-x)
Supplement: Supplementary file 1 — Additional file 1. Definitions of Obesity, The current smoking status, Alcohol abuse, Confirmed human influenza A or B, Comfirmed avian-origin A (H7N9), lymphocytopenia, Rhabdomyolysis, Acute kidney injury, Immunosuppression, Early antiviral therapy, The exposure to live poultry. [file 12879_2021_6485_MOESM1_ESM.doc]

Obesity was defined as body mass index（BMI） (weight in kg divided by the square of the height in meters) ≥30–39.9, and morbid obesity as BMI ≥40, excluding the pregnant woman. The current smoking status was recorded when a patient had smoked >10 cigarettes/day for at least 1 year preceding the study and had not given up until 1 month before the onset of illness. Alcohol abuse was considered if alcohol intake was more than three standard drinks per day and for more than 1 year. Confirmed human influenza A or B was defined as influenza-like illness with laboratory-confirmed human influenza A or B virus infection using Reverse Transcriptase-polymerase Chain Reaction （RT-PCR） test. Confirmed avian-origin A (H7N9) was defined as influenza-like illness or suspected patients when H7N9 virus infection was proved by RT-PCR. Lymphocytopenia was defined as lymphocyte count of <1500/mm^3^. Thrombocytopenia was defined as platelet count <150,000/mm^3^. Rhabdomyolysis was diagnosed based on the medical record review, which showed muscle pain or muscle weakness at the time of hospital admission; also, the and creatine kinase level was >10-fold upper limit of the normal in the patient. Acute kidney injury is defined as any of the following: increase in serum creatinine (SCr) by ≥0.3 mg/dL (≥26.5 µmol/L) within 48 h; increase in SCr ≥1.5-fold of the baseline, which might have occurred within the previous 7 days; urine volume <0.5 mL/kg/h for 6 h. Immunosuppression might have been caused by the presence of chemotherapy or radiotherapy within 1 month before the onset of illness or glucocorticoid therapy (the equivalent of 30 mg of prednisone/day) for 15 days before the onset of illness. Early antiviral therapy was considered when administered within 48 h from the onset of symptoms. The exposure to live poultry was defined as close contact with chicken or pigeons or visiting either a live poultry retailer or a market selling live poultry within 2 weeks before the onset of illness.
