# Supplementary material for: A comparison of demographic, epidemiological and clinical characteristics of hospital influenza-related viral pneumonia patients
Source: BMC Infect Dis. 2021 Sep 25;21:1002. doi: 10.1186/s12879-021-06485-x (PMC8466655; doi:10.1186/s12879-021-06485-x)
Supplement: Supplementary file 2 — Additional file 2. Table S1: Univariate Analysis of Factors Associated with Invasive Mechanical Ventilation due to Three Types Hospitalized Influenza-related Viral Pneumonia Patients. [file 12879_2021_6485_MOESM2_ESM.doc]

| Variables Non-invasive mechanical Invasive mechanical p-value  ventilation ventilation  (n=183) (n=32) |
| --- |
| Shortness of breaths, no. (%) 94 (51) 30 (94) <0.001  Neutrophils (%),  median (IQR) 77 (63.1–84.9) 87.7 (77.8–90.1) <0.001  C-reactive protein (mg/L),  median (IQR) 43.9 (14.6–93.4) 90.9 (38–126.2) 0.011  Lymphocytes <1500/mm^3^, no. (%) 142 (78) 32 (100) 0.006  Lymphocytes (/mm^3^),  median (IQR) 860 (480–1400) 450 (300–640) <0.001  Procalcitonin >0.5  ng/mL, no. (%) 30 (24) 17 (59) <0.001  Aspartate aminotransferase  >40 U/L, no. (%) 68 (38) 21 (66) 0.006  Aspartate aminotransferase (U/L),  median (IQR) 30 (20–52) 66.5 (33–103.8) <0.001  Creatine kinase >200 U/L, no. (%) 28 (16) 14 (47) <0.001  Lactate dehydrogenase  >250 U/L, no. (%) 105 (61) 29 (97) <0.001  D-dimer >700 μg/L, no. (%) 126 (71) 30 (94) 0.013  D-dimer (μg/L),  median (IQR) 1184 (671–2346) 3877 (1763.5–7436.5) <0.001  Blood urea nitrogen (mmol/L),  median (IQR) 4.9 (3.7–7.5) 9.2 (6–13) <0.001  PaO_2_:FiO_2_ (mmHg),  median (IQR) 240 (163.2–298.8) 133.3 (92.6–-197) <0.001  Positive bacterial culture (blood or sputum) on presentation or  during hospitalization, no. (%) 17 (9) 23 (72) <0.001  Positive bacterial culture (sputum) on presentation or during  hospitalization, no. (%) 16 (14) 20 (65) <0.001  Computed tomography consistent with  pneumonia at admission, no. (%)  Involvement of both lungs 156 (85) 32 (100) 0.018  Consolidation 105 (57) 30 (94) <0.001  CURB-65 score  ≥2, no. (%) 41 (24) 21 (66) <0.001  Pro-B-type natriuretic peptides (pg/mL),  median (IQR) 310 (94.5–719.5) 517 (272–1401) 0.052 |

Abbreviations: PaO_2_:FiO_2_, Partial pressure arterial oxygen/fraction of inspired oxygen, IQR, interquartile range
