# Supplementary material for: A comparison of demographic, epidemiological and clinical characteristics of hospital influenza-related viral pneumonia patients
Source: BMC Infect Dis. 2021 Sep 25;21:1002. doi: 10.1186/s12879-021-06485-x (PMC8466655; doi:10.1186/s12879-021-06485-x)
Supplement: Supplementary file 3 — Additional file 3. Table S2: Univariate Analysis of Factors Associated with Death due to Three Types Hospitalized Influenza-related Viral Pneumonia Patients. [file 12879_2021_6485_MOESM3_ESM.doc]

| Variables Non-death Death p-value  (n=197) (n=18) |
| --- |
| Respiratory frequency (breaths/min),  median (IQR) 20 (19–22) 22 (20–33) 0.001  Neutrophils (%),  median (IQR) 77.2 (65.8–86.5) 84.7 (77.8–93.4) 0.039  Hemoglobulin (g/dL),  median (IQR) 123 (105–135) 103.5 (96–115.3) 0.030  Platelets (/mm^3^),  median (IQR) 160000 (106000–249000) 116000 (55250–208000) 0.039  C-reactive protein (mg/L),  median (IQR) 46.4 (15.4–97.7) 97.1 (27.5–127.8) 0.043  Blood urea nitrogen (mmol/L),  median (IQR) 5.1 (3.8–8) 9.5 (4.8–13.9) 0.005  D-dimer (μg/L),  median (IQR) 1287.5 (684.8–2617) 3864 (1385–9198) 0.004  White blood cells count  >10,000/mm^3^, no. (%) 42 (21) 8 (44) 0.039  Procalcitonin >0.5  ng/mL, no. (%) 39 (27) 8 (57) 0.031  PaO_2_:FiO_2_—mmHg,  median (IQR) 215.6 (156.7–298.2) 135.8 (95.7–212.3) 0.004  Positive bacterial culture (blood or sputum) on presentation or  during hospitalization, no. (%) 29 (15) 11 (61) <0.001  Positive bacterial culture (sputum) on presentation or during  hospitalization, no. (%) 27 (21) 9 (64) 0.001  CURB-65 score  ≥2, no. (%) 51 (27) 11 (65) <0.001  Mechanical ventilation, no. (%)  Noninvasive 14 (7) 6 (33) 0.003  Invasive 22 (11) 10 (56) <0.001  Shock, no. (%) 6 (3) 5 (28) 0.001  Pro-B-type natriuretic peptides (pg/mL),  median (IQR) 310 (118.3–800.3) 559 (404–1865) 0.085 |

Abbreviations: PaO_2_:FiO_2_, Partial pressure arterial oxygen/fraction of inspired oxygen, IQR, interquartile range
