# Supplementary material for: A comparison of demographic, epidemiological and clinical characteristics of hospital influenza-related viral pneumonia patients
Source: BMC Infect Dis. 2021 Sep 25;21:1002. doi: 10.1186/s12879-021-06485-x (PMC8466655; doi:10.1186/s12879-021-06485-x)
Supplement: Supplementary file 4 — Additional file 4. Table S3: Multivariate Analysis of Factors Associated with Invasive Mechanical Ventilation due to Three Types of Hospital Influenza-related Viral Pneumonia Patients. [file 12879_2021_6485_MOESM4_ESM.doc]

| Variables OR (95% CI) p-value |
| --- |
| White blood cells count>10,000/mm^3^ 0.819  Hemoglobulin (g/dL) 0.791  Platelet count (/mm^3^) 0.524  C-reactive protein (mg/L) 0.137  Procalcitonin >0.5 ng/mL 0.120  Aspartate aminotransferase (U/L) 0.203  D-dimer >2100 μg/L 0.072  Positive bacterial culture (blood or sputum) 0.059  on presentation or during hospitalization  Positive bacterial culture (sputum) on 7.71 (2.48–24.03) <0.001  presentation or during hospitalization  Computed tomography consistent with  pneumonia at admission  Involvement of both lungs 0.071  Consolidation 13.67 (1.54–121.12) 0.019  Pro-B-type natriuretic peptides (pg/mL) 0.197 |

Abbreviations: OR, odds ratio, CI, confidence interval, IQR, interquartile range
